# Incidence and predictors of surgical site infection after distal femur fractures treated by open reduction and internal fixation: a prospective single‐center study

**DOI:** 10.1186/s12891-021-04132-9

**Published:** 2021-03-08

**Authors:** Chao Zhu, Junzhe Zhang, Junyong Li, Kuo Zhao, Hongyu Meng, Yanbin Zhu, Yingze Zhang

**Affiliations:** 1 Department of Orthopaedic Surgery, the Third Hospital of Hebei Medical University, No. 139 Ziqiang Road, Hebei Province 050051 Shijiazhuang, PR China; 2 Key Laboratory of Biomechanics of Hebei Province, Orthopaedic Research Institution of Hebei Province , Shijiazhuang Province Shijiazhuang, China; 3 Department of Orthopedics, the Affiliated Jiangning Hospital with Nanjing Medical University, Nanjing, China

**Keywords:** Predictor, Surgical site infection, Distal femoral fracture, Internal fixation surgery

## Abstract

**Background:**

There remain limited data on the epidemiological characteristics and related predictors of surgical site infection (SSI) after open reduction and internal fixation (ORIF) for distal femur fractures (DFFs). We designed this single-centre prospective study to explore and forecast these clinical problems.

**Methods:**

From October 2014 to December 2018, 364 patients with DFFs were treated with ORIF and followed for complete data within one year. Receiver operating characteristic (ROC) analyses, univariate Chi-square analyses, and multiple logistic regression analyses were used to screen the adjusted predictors of SSI.

**Results:**

The incidence of SSI was 6.0 % (22/364): 2.4 % (9/364) for superficial SSIs and 3.6 % (13/364) for deep SSIs. *Staphylococcus aureus* (methicillin-resistant *S. aureus* in 2 cases) was the most common pathogenic bacteria (36.8 %,7/19). In multivariate analysis, parameters independently associated with SSI were: Open fracture (OR: 7.3, *p* = 0.003), drain use (OR: 4.1, *p* = 0.037), and incision cleanliness (OR: 3.5, *p* = 0.002). An albumin/globulin (A/G) level ≥ 1.35 (OR: 0.2, *p* = 0.042) was an adjusted protective factor for SSI.

**Conclusions:**

The SSI after ORIF affected approximately one in 15 patients with DFFs. The open fracture, drain use, high grade of intraoperative incision cleanliness, and preoperative A/G levels lower than 1.35 were significantly related to increasing the risk of post-operative SSI after DFFs. We recommended that more attentions should be paid to these risk factors during hospitalization.

**Trial registration:**

NO 2014-015-1, October /15/2014, prospectively registered.

We registered our trial prospectively in October 15, 2014 before the first participant was enrolled. This study protocol was conducted according to the Declaration of Helsinki and approved by the Institutional Review Board. The ethics committee approved the Surgical Site Infection in Orthopaedic Surgery (NO 2014-015-1). Data used in this study were obtained from the patients who underwent orthopaedic surgeries between October 2014 to December 2018.

## Background

Distal femur fractures (DFFs) are relatively uncommon but severe in orthopaedic trauma, comprising approximately 8.7 % of all femoral fractures and 0.8 % of total body fractures in Chinese adults [1]. These fractures show a bimodal distribution. On the one hand, most of the fractures in younger patients result from high-energy injuries, which are usually open and comminuted fractures [2, 3]. On the other hand, fractures in older patients are caused by low-energy injuries, with a one-year mortality rate up to 13.4 % [4]. Furthermore, 50 % of the DFFs potentially involve articular surface [1], and operative intervention is the most common option for these patients such as open reduction and internal fixation (ORIF) or close reduction and internal fixation (CRIF). However, excessive soft tissue dissection during operation may impair the already damaged soft tissue, possibly leading to postoperative complications such as surgical incision site, knee dysfunction and bone union [5, 6].

Of these complications, surgical site infection (SSI) is a major challenge for most orthopaedic and trauma surgeons. SSIs are known to increase the length of stay by an average of 10 days and to cost the National Health Service (NHS) of the United Kingdom an estimated £700 million per year [7]. It has been reported that about 50 % of SSI cases can be avoided by the application of evidence-based prevention strategies [8]. Therefore, the identification of SSI-related predictors and screening of at-risk patients may propose a cost-effective and simple approach for prevention of SSI occurrence. Recently, most researches concerned SSIs of the hip, tibial plateau, and ankle [9–13].

However, studies on the epidemiological characteristics of SSIs after adult DFFs treated by ORIF were rare. The incidence of SSI after operations for DFFs has been showed to vary from 0.3 to 11.2 % [14], and most factors are not conclusive. Hoffmann et al. identified open injury and current smoking as associated risk factors [6]. Lu et al. added obesity and diabetes mellitus to the SSI-related predictors [15]. Moreover, all three studies above were retrospective and were collectively limited by untimely data selection owing to recall and response bias.

We conducted the prospective study for two objectives: first, to restudy the epidemiological characters of SSI after ORIF for adult DFFs; second, to recognize the SSI-related factors and their cutoff values. We hypothesized that the open fracture, cigarette consumption, and obesity were associated with increased DFF infection.

## Methods

### Study design

A prospective study was designed, and data were collected from patients who underwent ORIF for DFFs from October 2014 to December 2018. Administrative permissions (NO 2014-015-1) were acquired by our team to access the clinical patient data used in our research, which was granted by the Committee on Ethics and the Institutional Review Board of the Third Hospital of Hebei Medical University. All patients who were 18 years old and over with acute DFFs treated by ORIF were included in this study. The exclusion criteria were listed as following: age less than 18 years old, old fractures (> 21 days from earliest trauma), pathological fractures, and first treatment at other hospitals. Patients with open fractures or with multiple fractures were also involved to investigate their effect on SSIs. Preoperative infections of the open fractures were excluded, and the old fractures were also excluded for the different operative procedures such as bone grafting procedures. All enrolled patients had intraoperative antibiotic prophylaxis and were separated into two groups based on the occurrence of SSI. The case group was defined as patients with SSIs, and the control group involved patients who did not suffer from SSIs. The endpoint of the prospective study was any evidence of SSI obtained from telephone assessment, interview, or the clinical information one year after surgery.

### SSI definition

The diagnostic criteria for SSI within one year postoperatively were based on the definition of the CDC [8]. A superficial SSI only infiltrates the skin or subcutaneous tissue of the operation site. A deep SSI was identified if it satisfied one of the following conditions: infection through the deep fascia, persistent wound effusion or dehiscence, local abscess requiring focal debridement and implant replace or retrieve. All the wound exudates from the patients were collected with swabs and sent for causative agent culture and sensitivity. Any inpatient who commenced antibiotic treatment for wound problems (redness, swelling, hot pain) but did not conform the criteria of the deep SSI was classified as a superficial SSI, regardless of any microbiology results.

### Data collection and definition of variables of interest

All the data mentioned below were collected by five well-trained investigators. Investigators followed the patients closely by morning work rounds and reviewed patients’ clinical data. The suture site was observed by researchers starting from the first day after ORIF until hospital discharge. After discharge, patients who had not developed SSI were followed by telephone at 2, 4, 6 and 12 months after discharge. Patients with suspicious SSI were required to return for re-examination and etiologic diagnosis. We usually recalled the patients for the radiological and clinical periodic evaluation every half a year after hospital discharge. During the study period, detailed variables of interest were collected and divided into four aspects.

Demographic variables included age (18 to 45, 46 to 59, and ≥ 60 years), sex, height (m), weight (kg), location (rural, urban), cigarette consumption, alcohol consumption, diabetes mellitus, hypertension, cardiovascular disease and body mass index (BMI, kg/m^2^). BMI was split into five groups according to the Chinese reference criteria: underweight, < 18.5; normal, 18.5 to 23.9; overweight, 24 to 27.9; obesity, 28 to 31.9; and morbid obesity ≥ 32.

Fracture-related variables included injury type (closed, open), concurrent fracture sites (single fracture, multiple fractures), affected side,and injury mechanism. Injury mechanisms were divided into two groups: low-energy (fall from a standing height) and high-energy (falling accidents from high places, human violence and others).

Operation-related variables included history of previous operation at any site, preoperative stay, postoperative stay, intraoperative blood loss (< 400 and ≥ 400 mL), operation duration (< 120, 120 to 180, and > 180 min), anaesthetic type (local, combined spinal-epidural, and general), internal fixator use (plate or no plate), intraoperative drainage use, incision cleanliness (I, clean; II, potentially contaminated; and III, contaminated), and the American Society of Anesthesiologists (ASA, I-II and III-IV) classification system [9]. The incision cleanliness was evaluated before the operation, and the dirty/infected incision was excluded from the study. Preoperative stay was defined as the time period from the first injury to ORIF, which was separated into two groups: 1, < 7 days and 2, ≥ 7 days.

Laboratory-related variables were assessed within 24 h preoperatively and were conventionally divided into normal (reference range), above normal, and below normal. These biochemistry indices involved white blood cell (WBC) counts, and neutrophil granulocyte (NEUT), lymphocyte (LYM), monocyte (MON), eosinophil granulocyte (EOS), basophilic granulocyte (BAS), red blood cell (RBC), blood platelet (PLT), blood glucose (GLU), serum total protein (TP), albumin (ALB), globulin (GLOB), and albumin/globulin (A/G).

### Statistical analysis

Statistical analysis was conducted with SPSS version 25.0 (IBM Corp., Armonk, NY, USA). The continuous variables were showed as the median, mean ± standard deviation (SD). The distributions of all data were evaluated for normality by the Shapiro-Wilk test. A Whitney U test or t test was used to compare continuous variables between SSI and non-SSI groups according to the Shapiro-Wilk test. For the continuous variables with statistical significance (*p* < 0.05), receiver operating characteristic (ROC) analyses were carried out to identify the optimum cut-off value. Subsequently, categorical variables were compared by the chi-square test. Predictors with significance (*p* < 0.05) from the single factor analysis were entered into multiple logistic regression analyses (backward LR). In addition, surgical duration, diabetes mellitus, hypertension, cardiovascular diseases and BMI were selected into the model for the variables of interest. The odds ratio (OR) and 95 % confidence interval (95 % CI) were conducted to evaluate the correlation magnitude between factors and SSI risk. Normally a *p* < 0.05 was considered statistically significant.

## Results

### The selection of the patients

**Fig. 1:**
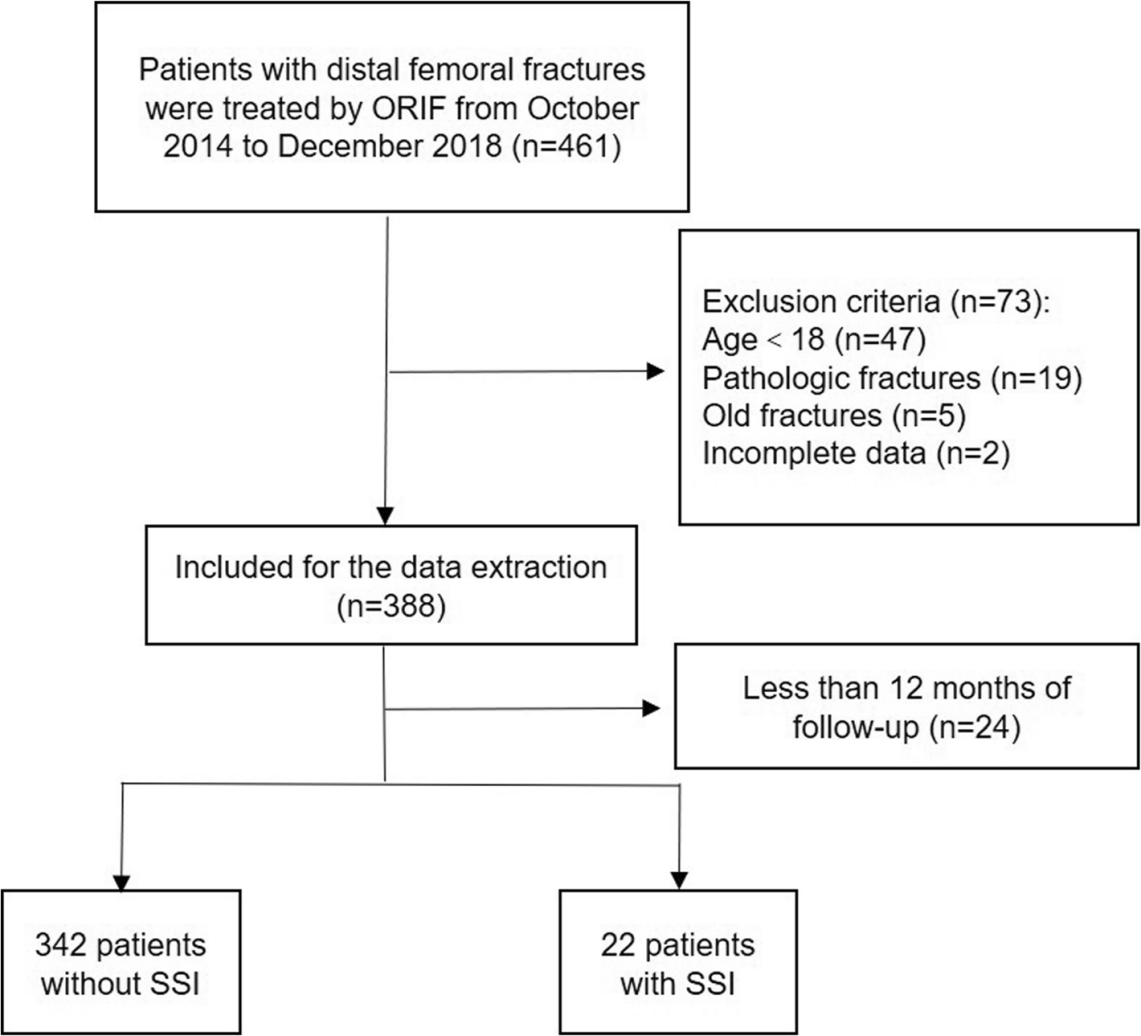
The flow chart for the selection of study participants

Figure 1 showed the flow chart for the selection of participants. A total of 461 patients with DFFs were admitted for ORIF, and 47 patients were less than 18 years old; 19 suffered from pathological fractures; 5 had old fractures; 2 had incomplete data, and 24 were lost (6.2 %) to follow-up. 364 patients were included for the final analysis.

### Diagnostic time points and incidence of postoperative SSI

**Fig. 2:**
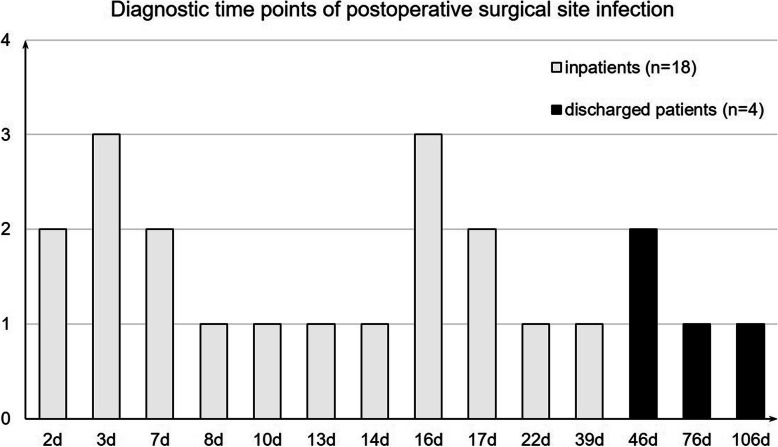
Diagnostic time points of postoperative surgical site infection

As shown in Fig. 2, the median time for diagnosis of SSI was 14 days after ORIF with a range from 2 to 106 days. The most of SSIs (81.8 %,18/22) was found during the subsequent hospitalizations. The total incidence of SSIs was 6.0 % (22/364). The superficial SSIs accounted for 2.4 % (9/364) and deep SSIs 3.6 % (13/364).

### Frequency of causative bacteria

Table 1 demonstrated the frequency of causative bacteria. A total of 31 wound swabs were sent for test, and 12 were negative. Of 22 patients with SSIs, 19 (86.4 %, 19/22) were successfully tested for causative bacteria. Swabs from the wounds were cultured for bacterial species as follow. *Staphylococcus aureus* (*methicillin-resistant Staphylococcus aureus* in 2 cases) was the most common (36.8 %,7/19), followed by mixed bacteria (31.6%, 6/19), *Escherichia coli* ( 21.1 %, 4/19), *Enterobacter cloacae* (21.1 %, 4/19), *Acinetobacter baumannii* (15.8 %, 3/19), *Pseudomonas aeruginosa* (15.8 %, 3/19), and others (Table 1).

**Table 1:** Frequency of causative bacteria

Bacteria	Frequency
**Single-bacteria causing SSI**	13
* Staphylococcus aureus*	2
*Pseudomonas aeruginosa*	2
*Enterobacter cloacae*	3
*Escherichia coli*	2
*Acinetobacter baumannii*	2
*Coagulase negative staphylococcus*	1
*Enterococcus faecalis*	1
**Mixed-bacteria causing SSI**	6
*Staphylococcus aureus + Staphylococcus epidermidis*	1
*Staphylococcus aureus + Enterococcus faecalis*	1
*Staphylococcus aureus + Klebsiella pneumoniae pneumoniae*	1
*Enterobacter cloacae + methicillin-resistant Staphylococcus aureus (MRSA)*	1
*Escherichia coli + methicillin-resistant Staphylococcus aureus (MRSA)*	1
*Escherichia coli + Acinetobacter baumannii + Enterobacter cloacae + Pseudomonas aeruginosa*	1

Abbreviation: *SSI* surgical site infection

### Patient demographic data and fracture characteristics

As shown in Table 2, for the 364 patients, the average age was 53.7 ± 17.0 years, and BMI was 25.6 ± 4.1 kg/m^2^. The median age of the three ordinal groups was 34, 51 and 68 years separately. The median BMI of the five ordinal groups was 18.0, 22.9, 25.4, 29.3 and 33.3 kg/m^2^ separately. The cohort included 193 males and 171 females, with 215 left-side and 149 right-side fractures. Furthermore, location (*p* = 0.041), smoking status (*p* = 0.004), alcohol consumption (*p* = 0.003), open fracture (*p <* 0.001), injury mechanism (*p* = 0.006), and concurrent fracture sites (*p* = 0.001) were demonstrated to correlate with SSI.

**Table 2:** Patient demographic data and fracture characteristics

Variables	All patients (*n* = 364)	Patients without SSI (*n* = 342)	Patients with SSI(*n* = 22)	*p* value
Gender (males),n(%)	193(53.0)	177(51.8)	16(72.7)	0.056^c^
Age (years), median	54	55	48	0.186^b^
Age (years)				0.192^c^
18–45, n(%)	111(30.5)	103(30.1)	8(36.4)	
46–59, n(%)	105(28.8)	96(28.1)	9(40.9)	
≥ 60, n(%)	148(40.7)	143(41.8	5(22.7)	
Location (rural), n(%)	262(72.0)	242(70.8)	20(90.9)	0.041^c^*
BMI (kg/m^2^), mean ± SD	25.6 ± 4.1	25.6 ± 4.2	26.1 ± 4.0	0.454^a^
Obesity (BMI ≥ 28), n(%)	70(19.2)	61(17.8)	9(40.9)	0.084^c^
Diabetes mellitus, n(%)	86(23.6)	81(23.7)	5(22.7)	0.918^c^
Hypertension, n(%)	95(26.1)	89(26.0)	6(27.3)	0.897^c^
Cardiovascular diseases, n(%)	54(14.8)	52(15.2)	2(10.5)	0.578^c^
History of previous operation, n(%)	115(31.6)	110(32.2)	5(22.7)	0.356^c^
Smoking, n(%)	50(13.7)	42(12.3)	8(36.4)	0.004^c^*
Alcohol consumption, n(%)	58(15.9)	49(14.3)	9(15.5)	0.003^c^*
Open fracture, n(%)	86(23.6)	69(20.2)	17(77.3)	< 0.001^c^*
Mechanism (high energy), n(%)	232(63.7)	212(62.0)	20(90.9)	0.006^c^*
Side (left), n(%)	215(59.1)	201(58.8)	14(63.6)	0.653^c^
Concurrent fractures (≥ 2 sites), n(%)	176(48.4)	158(44.2)	18(81.8)	0.001^c^*

Abbreviation: *SSI* surgical site infection, *BMI* body mass index.

^a^ Student t test.

^b^ Mann-Whitney U test.

^c^ Pearson Chi-Square test.

* indicates significant variable at *p* < 0.05.

### Details of operation‐related variables

As shown in Table 3, a significant difference was observed for the variables of postoperative stay (*p* < 0.001) between the two groups. Moreover, both the incision cleanliness (*p* < 0.001) and intraoperative drain use (*p* = 0.047) were demonstrated to correlate with SSI. In the 16 patients with intraoperative drains and postoperative SSIs, the mean duration of drainage was 4.1 ± 2.9 days and average drainage volume was 173.1 ± 115.6 mL. There were 12 patients who had surgical debridement due to SSIs. Only two patients underwent reoperations for implant exchange, and no patients required amputations.

**Table 3:** Details of surgical data

Variables	All patients (*n* = 364)	Patients without SSI (*n* = 342)	Patients with SSI(*n* = 22)	*p* value
Preoperative stay (days), median	6	6	5	0.184^b^
Preoperative stay (≥ 7 days), n(%)	176(48.4)	165(48.2)	11(50.0)	0.873^c^
Incision cleanliness				< 0.001^c^*
I, n(%)	333(91.5)	322(94.2)	11(50.0)	
II, n(%)	18(5.0)	14(4.1)	4(18.2)	
III, n(%)	13(3.5)	6(1.8)	7(31.8)	
ASA				0.133^c^
I-II, n(%)	236(64.8)	225(65.8)	11(50.0)	
III-V, n(%)	128(35.2)	117(34.2)	11(50.0)	
Anesthesia type				0.497^c^
Local anesthesia, n(%)	16(4.4)	14(4.1)	2(9.1)	
Combined spinal-epidural, n(%)	155(42.6)	147(43.0)	8(36.4)	
General, n(%)	193(53.0)	181(52.9)	12(54.5)	
Drainage use, n(%)	190(52.2)	174(50.9)	16(72.7)	0.047^c^*
Plate use(vs. without plate), n(%)	282(77.5)	263(76.9)	19(86.4)	0.303^c^
Intraoperative blood loss (ml), median	400	400	550	0.151^b^
Intraoperative blood loss ≥ 400 mL, n(%)	174(47.8)	161(47.1)	13(59.1)	0.274^c^
Surgical duration (minutes), median	165	163	180	0.136^b^
Surgical duration				0.312^c^
< 120 min, n(%)	58(15.9)	56(16.4)	2(9.1)	
120–180 min, n(%)	191(52.5)	181(52.9)	10(45.5)	
> 180, n(%)	115(31.6)	105(30.7)	10(45.5)	
Postoperative stay (days), median	12	11	36	< 0.001^b^*

Abbreviation: *SSI* surgical site infection, *ASA* American Society of Anesthesiologists

^b^ Mann-Whitney U test.

^c^ Pearson Chi-Square test.

* indicates significant variable at *p* < 0.05.

### Laboratory variables and the optimum cut‐off value

Table 4 depicted the univariate analysis of laboratory-related variables. A significant difference was observed for the variables of ALB level (*p* = 0.017) and A/G level (*p* < 0.001) between the two groups. Table 5 showed that ROC analysis was performed to identify the area under the curve and the optimum cut-off value for each statistically significant variable listed above. The cut-off values for ALB and A/G levels were 30.3 g/L and 1.35, respectively. Based on these cut-off values, we dichotomised the variables.

**Table 4:** Univariate analysis of laboratory-related variables

Variables	All patients (*n* = 364)	Patients without SSI (n = 342)	Patients with SSI(n = 22)	*p* value
WBC (10^9^/L)				0.420^c^
References (4–10), n(%)	208(57.1)	198(57.9)	10(45.5)	
> 10, n(%)	152(41.8)	140(40.9)	12(54.5)	
NEUT (10^9^/L)				0.476^c^
References (1.8–6.3), n(%)	142(39.0)	135(39.5)	7(31.8)	
> 6.3, n(%)	222(61.0)	207(60.5)	15(68.2)	
LYM (10^9^/L)				0.812^c^
References (1.1–3.2), n(%)	209(57.4)	195(57.0)	14(63.6)	
< 1.1, n(%)	154(42.3)	146(42.7)	8(36.4)	
MON (10^9^/L)				0.818^c^
References (0.1–0.6), n(%)	124(34.1)	117(39.5)	7(31.8)	
> 0.6, n(%)	240(65.9)	225(65.8)	15(68.2)	
EOS (10^9^/L)				0.829^c^
References (0.02–0.52), n(%)	265(72.8)	248(72.5)	17(77.3)	
< 0.02, n(%)	96(26.4)	91(26.6)	5(22.7)	
BAS (10^9^/L)				0.408^c^
References (0–0.06), n(%)	332(91.2)	313(91.5)	19(86.4)	
> 0.06, n(%)		29(8.5)	3(14.0)	
RBC (10^12^/L)				0.076^c^
References, n(%)	110(30.2)	107(31.3)	3(13.6)	
<Lower limit, n(%)	263(72.3)	244(68.7)	19(86.4)	
PLT (10^9^/L)				0.209^c^
References (125–300), n(%)	215(59.1)	206(60.2)	9(40.9)	
< 125, n(%)	22(6.0)	20(5.8)	2(9.1)	
> 300, n(%)	127(34.9)	116(33.9)	11(50.0)	
TP (< 65 g/L)	280(76.9)	264(77.2)	16(72.7)	0.630^c^
ALB(g/L), mean ± SD	33.8 ± 5.7	34.0 ± 5.6	30.5 ± 6.3	0.017^a^*
ALB (< 30 g/L), n(%)	103(28.3)	91(26.6)	12(11.7)	0.005^c^*
GLOB (< 20 g/L), n(%)	73(20.1)	71(20.8)	2(9.1)	0.294^c^
A/G, mean ± SD	1.5 ± 0.4	1.5 ± 0.4	1.2 ± 0.3	< 0.001^a^*
A/G				0.001^c^*
< 1.35, n(%)	168(46.2)	150(44.0)	18(81.8)	
≥ 1.35, n(%)	196(53.8)	192(56.0)	4(18.2)	
GLU (mmol/L)				0.766^c^
References (3.9–6.1), n(%)	160(44.0)	151(44.2)	9(40.9)	
> 6.1, n(%)	204(56.0)	191(55.8)	13(51.9)	

Abbreviation: *SSI* surgical site infection, *WBC* white blood cell, *NEUT* neutrophile, *LYM* lymphocyte, *MON* monocyte, *EOS* eosinophils, *BAS* basophilic, *PLT* platelet, *TP* total protein, *ALB* albumin, *GLOB* globulin, *A/G* albumin/globulin, *RBC* red blood cell, reference range(10^12^/L): females 3.5-5.0; males 4.0-5.5.

^a^ Student t test.

^c^ Pearson Chi-Square test.

* indicates significant variable at *p* < 0.05.

**Table 5:** The detailed results of the ROC curve

Variable	Cut-off value	Area under the curve (95 CI)	Sensitivity	Specificity	*p* value
ALB(g/L)	30.3	65.2 %(52.9 %-77.4 %)	73.0 %	59.1 %	0.017
A/G	1.35	71.8 %(62.0 %-81.6 %)	56.3 %	81.9 %	0.001

Abbreviation: *ROC* receiver operating characteristic, *ALB* albumin, *A/G* albumin/globulin

### Multiple logistic regression analysis

Of the 35 predictive variables listed above, 10 factors were correlated to SSI, which were location (*p* = 0.041), smoking consumption (*p* = 0.004), alcohol consumption (*p* = 0.003), open fracture (*p <* 0.001), injury mechanism (*p* = 0.006), concurrent fracture sites (*p* = 0.001), incision cleanliness (*p <* 0.001), drain use (*p* = 0.047), ALB level (*p* = 0.005) and A/G level (*p* = 0.001). Hence, these 10 factors were included in the multiple logistic regression model. Finally, Table 6 indicated that open fracture (*p* = 0.003), drain use (*p* = 0.037) and incision cleanliness (*p* = 0.002) were showed as independent risk factors of SSIs, while A/G level ≥ 1.35 (*p* = 0.042) an independent protective factor of SSIs. The Hosmer-Lemeshow test showed good fitness (*X*^2^ = 5.2; *p* = 0.735).

**Table 6:** Multivariate analysis of factors associated with SSI after ORIF

Variables	Odds ratio	95 % CI	*p* value
Open injury	7.3	2.0-26.7	0.003
Drainage use	4.1	1.1–15.5	0.037
Incision cleanliness	3.5	1.6–7.7	0.002
A/G ≥ 1.35	0.2	0.1-1.0	0.042

Abbreviation: *SSI* surgical site infection, *ORIF* open reduction and internal fixation, *CI* confidence interval, *A/G* albumin/globulin

## Discussion

Our present study of 364 patients indicated that the total incidence of SSI after ORIF for DFFs was 6.0 % with one-year follow-up, which was consistent with a retrospective multicentre analysis of 724 patients with a two-year follow-up [15]. We confirmed that *S. aureus* was the most frequently isolated pathogen of SSIs; patients with an SSI had a significantly longer postoperative stay than those without an SSI (45.5 vs. 15.5 days). After adjusting for confounding variables, open fracture, drain use, incision cleanliness and A/G level were significantly associated with SSIs.

Open fracture is a well-recognized risk factor for SSIs after orthopaedic surgeries [16]. In the present study, the odds ratio of open fracture was 7.3 in multivariate analysis models. The prevalence of SSI after open DFFs treated by ORIF was 19.8 % (17/86), which was in the range from 10.4 to 20.0 % according to previous studies on lower limbs [6, 12, 15]. However, Lu and his colleague reported that the prevalence of SSI after open intra-articular fractures of the distal femur treated by ORIF decreased to 10.4 %, which was approximately equal to half of the prevalence of our current study (19.8 %) [15]. In his study, all enrolled patients sustained an intraarticular fracture, such as femoral intercondylar fractures, and the blood supply around the intercondylar region was more abundant than that around the supracondylar region. An inadequate blood supply to this region could cause necrosis and/or insufficient wound healing. In addition, open fractures accounted for 9.4 % (68/724) in his study, while the proportion of open fractures was 23.6 % (86/364) in our study. Regarding open fractures with severe soft-tissue trauma, delaying ORIF might have a positive influence on preventing SSI. Damage control orthopaedics (DCO) facilitates the recovery of soft tissue, which provides sufficient control of the iatrogenic injuries [17]. All the open fractures and polytrauma patients were firstly conducted by aggressive debridement in our centre. Then, temporary traction bows or external fixators were performed according to the Gustilo-Anderson classification of soft tissue conditions at fracture sites and the time from trauma to surgical debridement [18]. Emergent one-stage procedures were performed for the acute Gustilo grade I and II fractures. Regarding Gustilo grade III fractures or Gustilo grade II fractures with the time from trauma to surgical debridement over 8 h, temporary traction bows or external fixators were performed for two-stage procedures.

Drains are used extensively in orthopaedics with the purpose of reducing the postoperative seroma. However, the criteria for using drains are not clear; patients often complain of anxious and pain from drainages, and drainage sites may retain a potential infection source. Some studies indicate that drain use plays a critical role in developing SSI after surgery [19, 20], which is in accordance with our results that an independent factor of drain use increased probability of SSI by 4.1 times (95 % CI, 1.1–15.5). Theoretically, drain use can increase the risk of infection. Bacteria, especially skin microbiome, spread along the drainage tube and have been identified from the tips of drainage tubes even as early as 48 h post-operatively [21]. Pennington et al. reported that long-term surgical drain retention was correlated with the risk of deep SSI after operations for degenerative spinal diseases [22].

It is well known that dirty surgical incisions have prolonged adverse impacts on wound closure. In the present investigation, we evaluated incision cleanliness during the operation. Then, we concluded a similar result that the risk of SSI increased by 3.5 (95 % CI, 1.6–7.7) times with every increase in the grade of incision cleanliness. In a retrospective case-control study that included 2617 cases of ankle fractures treated by ORIF, the OR of SSI was 1.8 (95 % CI, 1.1–3.2) with grade II-IV incision cleanliness, which is also in agreement with other reports of orthopaedic procedures [13, 23]. In clinical practice, medical staff examine surgical incisions and worry about infection in the early days after surgery [24]. However, patient-directed active surgical incision self-monitoring may help to further SSI reduction [25]. Hence, this finding would be a significant advance for SSI management that integrates reliable patients’ surgical incision surveillance into the clinical workflow.

The higher A/G level instead of ALB level was a significant independent protective factor for SSI after adult DFFs treated by ORIF, which was first reported in the present study, although a lower ALB level had been reported as a risk factor for SSIs after traumatic and elective orthopaedic surgeries [16]. Moreover, we further found that patients with A/G levels ≥ 1.35 had a 77.0 % decreased possibility of SSI when compared to those with A/G levels < 1.35. In the clinic, the A/G level (protein quotient) is the weight ratio of albumin to globulin, which is normal range from 1.2 to 2.4. The A/G maintains a lower level in protein deficiency or metabolic abnormalities. We proposed that the A/G level was more comprehensive and meaningful for predicting SSI risk than the ALB level. Both ALB and GLOB are necessary to maintain nutrition and immune balance for wound healing. On the one hand, ALB transports essential electrolyte and amino acids to improve the wound healing. On the other hand, GLOB, especially immunoglobulin, has indispensable and favourable anti-infection effects as well as lowers the high risk of postsurgical infection in the intensive care unit (ICU) [26–28].

The present study had three highlights: first, it was a prospective study with a one-year follow-up; second, ROC analysis was performed to detect a highly sensitive cut-off value for statistically significant continuous variables; and third, to our knowledge, it was first study to report that an A/G level ≥ 1.35 is an independent protective factor for SSI after adult DFFs treated by ORIF. However, the study was not without limitations. The old fracture might be an important variable, which were relatively uncommon (only 5 patients with old distal femur fractures were excluded) in our study. Temporary fixation of open fractures was usually accomplished with traction bows or external fixators in our center. However, we did not include the variable of the preoperative external fixation use. The interference of ORIF performed by multiple trauma surgeons was not excluded. In addition, some variables that potentially influence the development of SSI were not included, such as the internal fixation material (titanium or stainless) and surgical incision length.

## Conclusions

In summary, the overall incidence of SSIs after adult DFFs treated by ORIF was 6.0 % (22/364), with an incidence of superficial SSIs of 2.4 % (9/364) and of deep SSIs of 3.6 % (13/364). The open fracture, drain use, high grade of intraoperative incision cleanliness, and preoperative A/G levels lower than 1.35 were significantly associated with increasing the risk of SSI for DFFs after ORIF. We recommend careful assessment of these risk factors during hospitalization.

## Data Availability

The datasets used and analysed during the current study are available from the corresponding author upon reasonable request..

## Abbreviations

DFFs: Distal femur fractures
SSI: Surgical site infection
ORIF: Open reduction and internal fixation
ROC: Receiver operating characteristic
A/G: Albumin/globulin
BMI: Body mass index
